# Factors Associated With Pupil Toilet Use in Kenyan Primary Schools

**DOI:** 10.3390/ijerph110909694

**Published:** 2014-09-17

**Authors:** Joshua V. Garn, Bethany A. Caruso, Carolyn D. Drews-Botsch, Michael R. Kramer, Babette A. Brumback, Richard D. Rheingans, Matthew C. Freeman

**Affiliations:** 1 Department of Epidemiology, Rollins School of Public Health and Laney Graduate School, Emory University, Atlanta, GA 30322, USA; E-Mails: jgarn@emory.edu (J.V.G.); cdrews@emory.edu (C.D.D.-B.); mkram02@emory.edu (M.R.K.); 2 Department of Behavioral Sciences and Health Education, Rollins School of Public Health, Emory University, Atlanta, GA 30322, USA; E-Mail: bcaruso@emory.edu; 3 Department of Biostatistics, University of Florida, Gainesville, FL 32611, USA; E-Mail: brumback@phhp.ufl.edu; 4 Department of Global and Environmental Health, University of Florida, Gainesville, FL 32611, USA; E-Mail: rrheing@epi.ufl.edu; 5 Department of Environmental Health, Rollins School of Public Health, Emory University, Atlanta, GA 30322, USA

**Keywords:** sanitation, school sanitation, latrine use, toilet use, pupil to latrine ratio, pupil to toilet ratio, cleanliness, Kenya

## Abstract

The purpose of this study was to quantify how school sanitation conditions are associated with pupils’ use of sanitation facilities. We conducted a longitudinal assessment in 60 primary schools in Nyanza Province, Kenya, using structured observations to measure facility conditions and pupils’ use at specific facilities. We used multivariable mixed regression models to characterize how pupil to toilet ratio was associated with toilet use at the school-level and also how facility conditions were associated with pupils’ use at specific facilities. We found a piecewise linear relationship between decreasing pupil to toilet ratio and increasing pupil toilet use (*p* < 0.01). Our data also revealed significant associations between toilet use and newer facility age (*p* < 0.01), facility type (*p* < 0.01), and the number of toilets in a facility (*p* < 0.01). We found some evidence suggesting facility dirtiness may deter girls from use (*p* = 0.06), but not boys (*p* = 0.98). Our study is the first to rigorously quantify many of these relationships, and provides insight into the complexity of factors affecting pupil toilet use patterns, potentially leading to a better allocation of resources for school sanitation, and to improved health and educational outcomes for children.

## 1. Introduction

The problem of inadequate sanitation is important for school-aged children, who experience over 2.8 billion cases of diarrhea annually [1], and who bear much of the burden of soil-transmitted helminth morbidity [2]. Inadequate sanitation can lead to a number of health problems, including stunted growth [3,4], diarrheal illness [5,6,7], and even death [8,9]. Equitable access to school sanitation is of particular concern. Data are scarce, but recent estimates suggest that only 45% of schools in low income countries have adequate sanitation facilities [10].

The health and educational benefits of increasing the number of latrines in schools are still not well understood. To our knowledge, no trial assessing only the benefit of additional latrines in schools has been conducted, likely because implementing sanitation without hygiene is not seen as best practice and may not be policy relevant [11]. The only comprehensive school WASH trial that also included latrine provisions found decreased pupil absence, increased enrollment, and decreased diarrheal illness, but only among certain subsets of the study population [12,13,14], and found reduced helminth infection rates for the *Ascaris lumbricoides* worm, but not other helminths [15]. Furthermore, pupils attending schools in an arm that received latrine provisions received little benefit compared to pupils in otherwise similar intervention arms but without latrine provisions [12,13,14] and latrine provisions were even associated with increased pupil hand contamination [16]. These results suggest that other factors, besides simply providing school latrines, are important to the success of school sanitation interventions at scale.

One possibility for the mixed success of this previous trial is that while the number of latrines increased, latrine dirtiness could actually increase pupils’ exposure to disease [17]. For instance, studies have found that dirty school sanitation facilities are associated with increased bacterial pathogens throughout the bathroom [18], and with increased incidence of diarrhea [17], vomiting [17], and dysentery outbreaks [19]. Decreasing the pupil to latrine ratio in a school is hypothesized to improve the overall latrine use in that school [16,20], but for this increase in latrine use to improve public health, it must also coincide with a net reduction in pupils’ exposures to pathogens. 

Another possibility for the mixed success of this previous trial relates to the actual use of the latrines. There has been considerable attention to the child-centered design of sanitation facilities in schools [21,22], however little empirical data exist on how the type, design, and maintenance of facilities affect behavior or health. Provisions of toilets at schools do not guarantee that those toilets are used or are well maintained. When latrines are available, children may choose to use the latrines or urinals, to openly defecate or urinate in or around the school grounds, or to hold their use until they can access a preferable toilet or openly defecate outside of school [23]. Open defecation, which affects pupil health by increasing exposure to fecal pathogens, has been observed in lower resource schools even when school toilets are present [24]. Toilet avoidance behavior also occurs [25,26,27,28], and can affect pupil health by causing personal discomfort and even bowel or urinary problems [26,27]. However, no rigorous studies have quantified how characteristics of toilets that lead to improved use.

The purpose of this study was to quantify how school sanitation conditions are associated with pupils’ use of sanitation facilities in 60 primary schools in Kenya. We characterize how varying pupil to toilet ratio was associated with the overall use of toilets at schools. We also characterize how toilet conditions, such as toilet cleanliness, age, type, and structure were associated with pupils’ use at specific facilities.

## 2. Methods 

This study took place in 60 schools (17,564 pupils at baseline) from the Rachuonyo (*N* = 33), and Kisumu East/Nyando (*N* = 27) Districts in Nyanza Province, Kenya. Our study uses data gathered during a trial that was designed to understand if low-cost and easily implemented latrine cleaning supply and handwashing interventions decreased school absence [29]. Schools were randomized into three different arms: (1) a latrine cleaning arm, which received soap for handwashing, cleaning supplies for latrines, and training on maintenance, (2) a handwashing arm, which received soap only, and (3) a control arm, which received no intervention. All of the inputs in the intervention arms were provided after the baseline visit. Depleted or missing supplies were replenished to intervention schools as needed during the surveillance period, starting after the August school break and continuing to just prior to the final data collection. Upon completion of the study, the control arm received all the same inputs as the latrine cleaning arm. We used the data that were gathered in an observational setting, considering the toilet facility’s actual cleanliness (and other facility characteristics) without regard to whether or not a school was randomized to receive or actually had latrine cleaning supplies.

### 2.1. Data Collection 

All data for this study were collected by trained enumerators from the Great Lakes University of Kisumu. Data collection was conducted from late May 2010 through early November 2010. At each of five study rounds, enumerators observed sanitation conditions at each latrine and each urinal immediately upon arrival at the school in the morning. School visits were unannounced and on a randomly selected day during a given week within the study round period. These data were recorded using Syware Visual CE v10 software (Cambridge, MA, USA) on Dell Axim x51 (Round Rock, TX, USA) personal digital assistants. Enumerators recorded the latrine’s or urinal’s cleanliness (*i.e.*, ‘clean’, ‘slightly dirty’, ‘very dirty’), the presence of visible feces (*i.e.*, ‘no visible feces’, ‘small amounts of visible feces’, ‘feces very visible’) or visible urine (*i.e.*, ‘no visible urine’, ‘small amounts of visible urine’, ‘urine puddling’), the smell (*i.e.*, ‘minimal smell’, ‘strong smell inside’, ‘strong smell inside and outside’), the presence of flies (*i.e.*, ‘none’, ‘some flies inside’, ‘many flies inside’), and the presence of functioning shutters. The above three-level variables were re-categorized with the worst category being compared to the combined moderate and best category. This categorization was used in all analyses, and was chosen for simplicity of model interpretation. Because these variables were subjective to the enumerators, baseline measures were independently collected from two different enumerators at each toilet on the same day, and the inter-rater reliability was calculated (Appendix, Table A1). Only those variables with substantial inter-rater agreement, as defined by Landis and Koch to mean a Cohen’s kappa statistic of over 0.6 [30] were included in the final analyses. Although we collected data on sanitation conditions at the toilet-level, for most analyses we aggregated the variables to the level of the “block” or “toilet facility”, in order to better relate these predictors to pupils’ use of facilities. We use the terms “block” and “toilet facility” synonymously to mean a structure that contains any number of conjoined, similarly constructed toilets, which is typically assigned to either boys or girls for use.

Pupils’ use of toilet facilities was also observed at these corresponding five school visits. Observations of toilet use always took place during the 30-minute morning break, between 11:00 and 11:30 AM across all schools, and always took place after the observation of toilet facilities. Pupils’ toilet use was recorded on paper surveys by two trained enumerators who, from a discrete distance, tallied the number of pupils who approached and/or entered the block during the break period. It was not possible to observe the actual entrance into every individual toilet, because entrances were often on opposite sides of a given block. For this reason, toilet use was tallied at the block-level, rather than at the individual toilet-level; when the block only had one latrine—which was observed 36% of the time—then the block-level was also the toilet-level. However, the block-level is of interest, as it is the level of implementation of newly built groups of latrines or urinals. Because old, out-of-use toilets often remain standing, we limited all analyses to toilets that were actually used by pupils in grades 1–8, or indicated as in use by teachers at the school. 

The type of toilet facility was recorded on paper at the first and final round only. Enumerators recorded whether the toilet was a traditional latrine, a ventilated improved pit latrine (VIP: a latrine with a pipe from the pit to the top of the latrine, which is covered with a fly screen at the top of the outlet), a prefabricated plastic latrine, an above ground vault composting latrine, or a urinal. There was substantial agreement between measures at the two visits (kappa = 0.76), with the primary source of disagreement being between VIP latrines and traditional latrines that were classified differently at the two visits (probably due to a missing fly screen or broken pipe). Above ground vaults were uncommon (1%). We created an ‘uncertain/other’ category for the previously mentioned toilets that were either difficult to categorize across the two visits or uncommon. During the first and final visit, the enumerators also observed whether or not the toilet was installed by the SWASH+ trial—a trial that installed many new toilets between 2007 and 2008—and this information was used as a proxy for newer toilet age. We do not have any other information on whether other new latrines were constructed besides those built by SWASH+.

Total and sex disaggregated school enrollment were collected during the first and final rounds using school records, and these enrollment totals, along with the number of working latrines and urinals, were used to calculate the pupil to toilet ratios, separately for boys and girls. We calculated the pupil to toilet ratio at each time point, allowing for slight changes if either the enrollment or if the number of in use toilets varied over time. We also used the enrollment numbers to create a school enrollment variable, where we categorized schools into enrollment quartiles.

To further capture important confounders, we collected data on community characteristics in the areas around each school. Enumerators conducted interviews with the heads of household at 25 systematically sampled households in the catchment area of each school. Enumerators collected both observed and head-of-household-reported information on wealth, and WASH conditions in the household. These data were then aggregated for use as community-level variables in our analyses. We used latrine coverage (percent of households with a latrine), and the wealth index score (a continuous variable constructed using principal component analysis) [31] as markers of latrine availability outside of school and of socio economic status, respectively.

### 2.2. Analysis 

#### 2.2.1. School and Facility Characteristics 

We show descriptive statistics for the schools and toilet facilities under study. School-level data were aggregated across the five time points by taking the mean of the five follow-up values for a given school. Using this aggregated data, we report the mean and distribution among all 60 schools. Facility-level data are shown at the baseline visit. We show toilet use at both the school, and facility level. 

#### 2.2.2. Pupil to Toilet Ratio and School-Level Toilet Use

**Figure A1:**
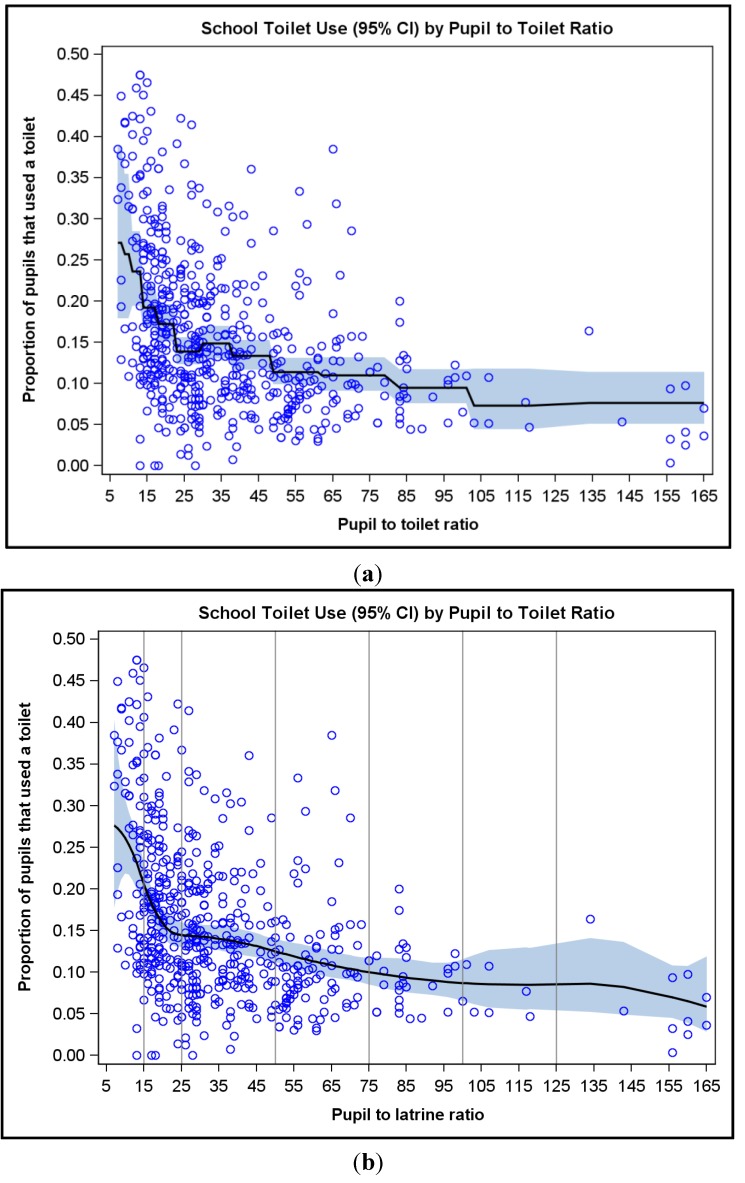
(**a**) Proportion of pupils who used a toilet at each school as a function of pupil to toilet ratio, fit with an ordinal pupil to toilet ratio variable. (**b**) Proportion of pupils who used a toilet at each school as a function of pupil to toilet ratio, fit with a piecewise quadratic spline (knots shown by vertical lines).

We used a multivariable logistic mixed effects model with a binomial outcome to characterize the relationship between pupil to toilet ratio and toilet use. The effect of pupil to toilet ratio was modeled as piecewise linear, with the locations of the knots (*i.e.*, breakpoints) being determined during exploratory graphical analyses (Appendix, Figure A1). Pupils’ toilet use was measured as the total pupil uses at a school during the 30 min break divided by the number of pupils at the school, by sex. Pupil to toilet ratio, calculated separately for each sex, was our primary predictor variable of interest. We report the adjusted odds ratio (aOR) comparing the odds of toilet use at various levels of pupil to toilet ratio, controlling for all the other variables in the model. Confounders were chosen *a priori* based on biological plausibility and from the very small existing literature on this topic. In preliminary analyses, interaction between sex and pupil to toilet ratio was assessed by including product terms in the model. However, we had predetermined that only interaction terms that were statistically significant with a p-value (*p*) of < 0.1 would persist in the final model, and by this criterion all interaction terms were excluded from our final model. Specifically, the model that we used was:

(1) $$\text{logit}\left({\text{μ}}_{it}\right)={\text{α}}_{0}+{\text{β}}_{1}{\text{Pupil to latrine ratio}}_{it}+{\text{β}}_{2}\left({\text{Pupil to latrine ratio}}_{it}-25\right){\text{X}}_{it}+\overset{Q}{\underset{q=1}{\sum }}{\text{γ}}_{q}{\text{ Confounders}}_{it}\;+{\text{ u}}_{0i}\;,$$

where μ*_it_* is the expectation of the response variable (school toilet use). The outcome and predictors were observed at the t^th^ round, in the i^th^ school for each sex attending the school. β_1_ represents the change in the log-odds of toilet use for each one unit increase in pupil to toilet ratio for schools with a pupil to toilet ratio of ≤ 25:1 (adjusting for all the other variables in the model). X*_it_* is a dummy variable that equals zero if the pupil to toilet ratio is ≤ 25 and equals one if pupil to toilet ratio is > 25. β_1_ + β_2_ represents the change in the log-odds of toilet use for each one unit increase in pupil to toilet ratio for schools with a pupil to ratio of > 25:1. u_0*j*_ represents a random intercept for each school. Confounders included sex, toilet coverage in the surrounding community, school enrollment quartiles, wealth index score, geographic district, and study round. 

We used this model to predict the increase in toilet use given the theoretical addition of one or more toilets at a school of a given enrollment size. We assume a variety of initial pupil to toilet ratios but, for simplicity, we always assumed an enrollment of 150 boys and 150 girls—numbers markedly similar to the population averages of our study population. The results might be interpreted as *if we were to add one sex-specific toilet to a school with a given sex-specific pupil to toilet ratio (e.g., 150:1, 75:1, 25:1, etc.) and with enrollment of 150 pupils of that sex, then the relative odds of toilet use would increase by ‘aOR’ times.*

**Figure 1:**
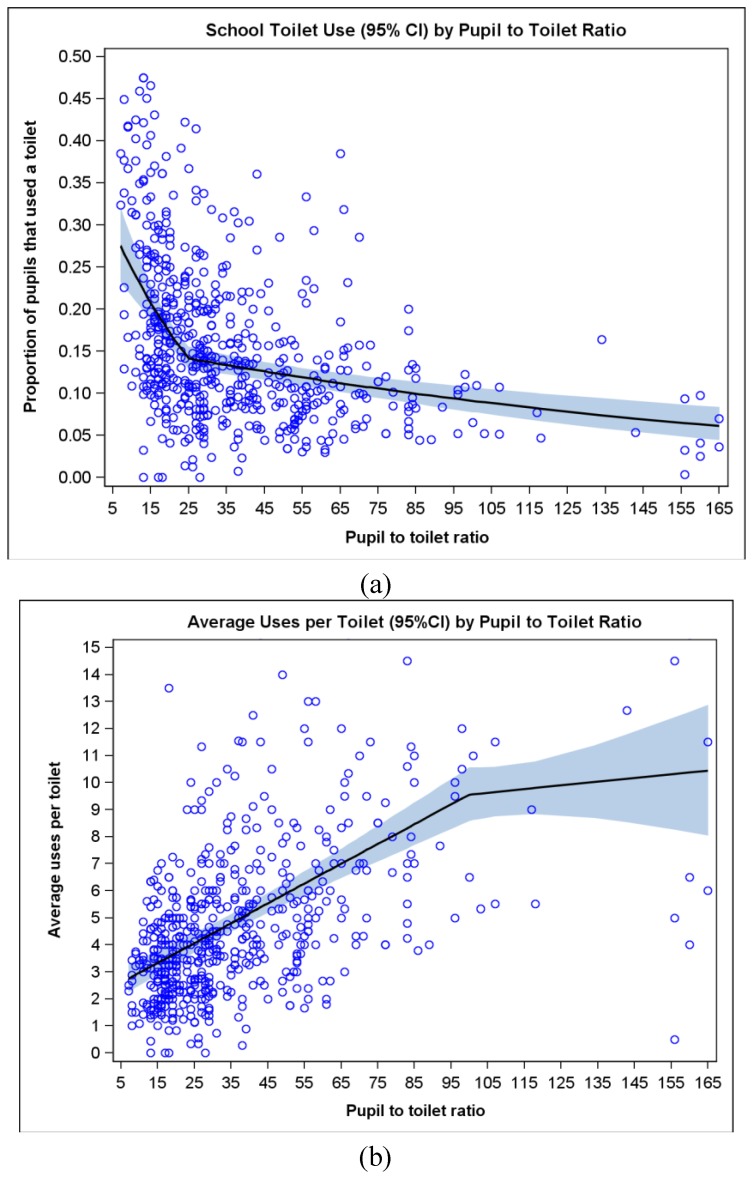
(**a**) Proportion of pupils who used a toilet at each school as a function of pupil to toilet ratio. (**b**) Average uses per toilet at each school as a function of pupil to toilet ratio. Pupil to toilet ratio was calculated separately for boys and girls. Both figures were fit with a piecewise trend line.

We used the logistic link (which produces an odds ratio), because models did not converge with the log link (which produces a risk ratio). We did not use linear regression, as it is suboptimal to model proportions that have values near zero, where the relationship is not linear. We used a simple linear spline with a single breakpoint due to its simple interpretation and as it fit the data well (Figure 1; Appendix, Figure A1).

For all of our regression models, we accounted for correlation of the repeated measures over time and for correlation of observations within schools [32]. All analyses were performed in SAS 9.3 (SAS Institute, Cary, NC, USA). We accounted for the correlation between repeated measures by specifying the R correlation matrix using the GLIMMIX procedure. We chose a compound symmetric covariance structure and also verified that this was an appropriate option using the robust ‘empirical’ option. Observations within schools were also correlated, and we accounted for this correlation by including a random intercept for school.

#### 2.2.3. Toilet Facility Characteristics Associated with Facility-Level Toilet Use 

We used a multivariable negative binomial mixed effects model to characterize the relationship between different toilet facility characteristics and the count of uses at specific facilities. The unit of analysis was the block or facility—a group similarly constructed and conjoined latrines/urinals. Pupils; use, measured at the facility, was the dependent variable, and that block’s characteristics were the predictors. We report the adjusted incidence rate ratio (aIRR) for each predictor. The aIRR compares the count of pupil uses during the 30-minute break between a toilet facility with the risk category and a toilet facility with the referent category, all other variables in the model being held constant. Interaction between sex and each of the other primary exposures of interest was assessed by including product terms in the model. Interaction between facility shutters and toilet type (urinal* vs.* latrine) was also assessed, as urinals are often built purposefully without shutters and were hypothesized to be different than latrines. We predetermined that only interaction terms that were statistically significant with a *p* of < 0.1 would persist in the final model. 

The general form of our adjusted model was:

(2) $$\text{log}\left({\text{μ}}_{ijt}\right)={\text{α}}_{0}+\overset{P}{\underset{p}{\sum }}{\text{β}}_{p}{\text{ Facility characteristics}}_{ijt}+\overset{Q}{\underset{q=1}{\sum }}{\text{γ}}_{q}{\text{ Confounders}}_{ijt}+\overset{R}{\underset{r=1}{\sum }}{\text{δ}}_{r}{\text{ Interaction terms}}_{ijt}+{\text{u}}_{0j}\;,$$

where μ*_ijt_* is the expectation of the response variable (count of uses at facilities). The outcome and predictors were observed at the t^th^ round, on the i^th^ toilet facility, which is in the j^th^ school. The facility characteristics included the facility’s cleanliness, age, presence of many flies, presence of shutters, number of toilets (using indicator variables), and type (VIP latrines, prefabricated plastic latrines, uncertain/other latrines, urinals, and traditional latrines as the referent,). Confounders included sex designation of the block, pupil to toilet ratio at the school, toilet coverage in the surrounding community, school enrollment quartiles, wealth index score, geographic district, and study round. Only the sex* cleanliness and the shutters *toilet type interaction terms met the criterion to be included in our final model. u_0*j*_ represents a random intercept for each school. We accounted for correlated data by specifying the working correlation matrix using the GLIMMIX procedure, as discussed previously.

## 3. Results 

### 3.1. School and Toilet Facility Characteristics 

We aimed to collect observations at 60 schools over five time points (300 observations), but due to school sporting events and holidays, we only collected complete data on 290 school observations (97%). An average of 301 pupils were enrolled per school, 48.6% of whom were girls (Table 1). The mean number of toilets at each school was 9.8—5.0 of these being designated for boys and 4.8 being designated for girls. The median pupil to toilet ratio was 29 for boys (range: 11–129) and 30 for girls (range: 8–159). 60.6% of the schools reported having a water source, 88.3% had water available for cleaning, and 25.2% had supplies available for toilet cleaning. Surveys of households in the catchment areas around the schools revealed that on, average, 58.3% of the households had a working latrine.

**Table 1:** School demographics (*N* = 60 schools), aggregating 5 follow-up measures. *

Variable	Mean or % (SD)
Pupils enrolled per school^‡^	301.4 (166.7)
Percentage of girls per school	49% (4)
Pupil to toilet ratio for boys^‡^	37.0 (24.0)
Pupil to toilet ratio for girls^‡^	36.6 (24.6)
Number of toilets per school	9.8 (3.9)
Number of designated boy toilets per school	5.0 (2.0)
Number of designated girl toilets per school	4.8 (2.4)
Percentage of households in surrounding community with working latrines	58% (20)
Percentage of schools with a water source	61% (38)
Percentage of schools with water available for toilet cleaning	88% (20)
Percentage of schools with supplies for latrine cleaning	25% (33)
Percentage of pupils in school that used a toilet during the 30 minute break	16% (6)
Percentage of boys in school that used a toilet	15% (6)
Percentage of girls in school that used a toilet	17% (7)

* The 5 follow-up values were averaged together for each school, and the distributions of those average school-values are shown here. ^‡ ^Data are skewed. Median enrollment was 258.8 (range: 94.2–830.4). Median pupil to toilet ratio for boys was 29 (range: 11–129) and median pupil to toilet ratio for girls was 30 (range: 8–159).

On average, 15.7% of the pupils within each school used a toilet during the break; use was similar for boys (15.0%) and girls (16.6%; Table 1). The average toilet facility was used 8.1 (SD 8.5) times per 30-minute break (Table 2). We did not observe use at the individual toilet-level, but knowing the total uses per school and the number of latrines per school we were able to calculate that each latrine was used on average by four pupils during the 30-minute break. 

At each round, we observed an average of 258 toilet facilities and 594 latrines/urinals. Each block had an average of 2.3 toilets (range: 1–10; Table 2). Sanitation facilities varied in type, with 17.9% being traditional pit latrines, 39.5% ventilated improved latrines, 19.4% prefabricated plastic latrines, 14.0% were classified as uncertain or other types of latrines, and 8.5% were urinals. As for toilet conditions, 31.8% of the latrine facilities were observed to be very dirty, 12.8% had feces that were very visible, 8.9% had puddling urine, 19.4% had no shutter on the majority of toilets in the block, 10.0% had many flies inside, and 25.6% were had a strong smell both inside and outside the facility.

**Table 2:** Toilet facility conditions at baseline visit.

Variable	N (%) or Mean (SD)
Total number of toilet facilities	258 (100%)
Mean toilets per facility *	2.3 (1.4)
Mean pupil use per toilet facility *	8.1 (8.5)
Toilet facility conditions ^‡^	
‘Very dirty’	82 (31.8%)
‘Feces very visible’	33 (12.8%)
‘Most visible urine’	23 (8.9%)
‘Many flies inside’	27 (10.5%)
‘No shutter’	50 (19.4%)
‘Strong smell inside and outside’	66 (25.6%)
Newer age ^§^	128 (49.6%)
Number of toilets per facility	
1 toilet	92 (35.7%)
2 toilets	76 (29.5%)
3 toilets	46 (17.8%)
4 toilets	27 (10.5%)
5 toilets	6 (2.3%)
6 or more toilets	11 (4.3%)
Type of toilet facility	
Traditional latrine	45 (17.9%)
Ventilated improved pit latrine	102 (39.5%)
Prefabricated plastic latrines	50 (19.4%)
Uncertain/other ^||^	36 (14.0%)
Urinal	22 (8.5%)
Facilities assigned to girls	117 (46.6%)

* Data were skewed. Median latrines per block was 2 (range: 1–10). Median use per block was 6 (range: 0–55). ^‡ ^The worst category is shown, and the combined moderate and best category are the reciprocal. ^§ ^Whether the toilet facility was from SWASH+ served as a proxy for newer toilet age. ^|| ^The uncertain/other category primarily consists of VIP latrines that were not easily categorized (e.g., missing a fly screen/broken pipe).

The cleanliness variable captures several important aspects of pupils’ exposure to human excrement. For example, 98.2% of the time when a latrine block was observed to have the ‘most feces’ and 80.5% of the time when a latrine block was observed to have ‘puddles of urine’, that latrine block was also marked as being ‘very dirty’ (data not shown). Because the substantial correlation between these variables, only the cleanliness variable was used in adjusted analyses. On average during each of the five rounds, 635 pupils across the 60 schools used a toilet facility that was observed as being very dirty during the break (data not shown). 

### 3.2. Factors Associated with Latrine Use 

#### 3.2.1. Pupil to Toilet Ratio and School-Level Toilet Use

As pupil to toilet ratio increases (becomes worse) there is a linear decrease in pupil toilet use with a natural breakpoint (change in slope) at a pupil to toilet ratio of 25:1 (Figure 1a). However, as pupil to toilet ratio increases, the number of average uses per toilet also increases linearly up until a pupil to toilet ratio of 100:1 (Figure 1b), after which the average number of uses per toilet plateaus at between nine and 10 uses per toilet (*i.e.*, each toilet being used about once every 3–4 min throughout the entire 30 min break). In adjusted analyses, the predicted change in the log-odds of a pupil using a toilet for each one unit increase in pupil to toilet ratio was −0.030 (95% confidence interval (CI): −0.045, −0.014, *p* < 0.01), and that slope persisted up to a pupil to toilet ratio of 25:1, after which the slope (*i.e.*, β_1_ + β_2_) was −0.005 (95% CI: −0.007, −0.003, *p* < 0.01). 

We used our model based estimates to predict the increase in school toilet use given the theoretical addition of one or more sex-specific toilets, at a school of an initial sex-specific pupil to latrine ratio and enrollment size (Table 3). Although we previously found the slope between use and pupil to toilet ratio to be flatter for pupil to toilet ratios ≥ 25, the predicted relative increase in the odds of toilet use from adding one, or several, toilets is much greater for the schools with a lower initial pupil to toilet ratio. 

**Table 3:** Contrast of adjusted odds ratio for school toilet use and school pupil to toilet ratio, given additional toilets added to a school.

Starting Sex-Specific Pupil: Toilet Ratio	Hypothetical Addition of Toilets, for a Given Sex of Pupils	New Sex-Specific Pupil: Toilet Ratio *	Predicted Increase in Use aOR ^‡^ (95% CI)
15:1	+ 1 toilet	13.6:1	1.04 (1.02–1.06)
25:1	+ 1 toilet	21.4:1	1.11 (1.05–1.18)
50:1	+ 1 toilet	37.5:1	1.06 (1.03–1.10)
75:1	+ 1 toilet	50:1	1.13 (1.07–1.20)
150:1	+ 1 toilet	75:1	1.45 (1.21–1.74)
25:1	+ 5 toilets	13.6:1	1.40 (1.18–1.67)
50:1	+ 5 toilets	18.8:1	1.36 (1.23–1.51)
75:1	+ 5 toilets	21.4:1	1.43 (1.27–1.61)
150:1	+ 5 toilets	25:1	1.86 (1.38–2.51)
150:1	+ 10 toilets	13.6:1	2.61 (1.91–3.57)

* Assuming a school enrollment size of 150 boys/girls. The aOR compares the odds of school toilet use during the 30-minute break between schools with varying pupil to toilet ratios, all other variables in the model being held constant. ^‡ ^Model adjusts for sex, school enrollment quartiles, toilet coverage in the community, wealth index score, geographic district, and study round (also accounts for correlation between repeated measures and clustering within schools).

For example, the aOR from hypothetically adding one toilet for a given sex of pupils is 1.45 (95% CI: 1.21–1.74) in a school with a starting pupil to toilet ratio of 150:1, whereas it is only 1.04 (95% CI: 1.02–1.06) in a school with a starting pupil to toilet ratio of 15:1. The predicted odds of toilet use increases 2.61 fold (95% CI: 1.91–3.57) by hypothetically adding ten toilets for a given sex of pupils in a school with an initial sex-specific pupil to toilet ratio of 150:1. A number of other contrasts of policy interest are also shown.

#### 3.2.2. Toilet Facility Characteristics Associated with Facility-Level Toilet Use

A number of facility characteristics were associated with toilet use (Table 4). We found an interaction in how dirty toilet facilities were used, based on whether the facility was designated for boys or girls (*p* = 0.10), and there was some evidence, although our estimate was imprecisely measured, that dirtiness may be a deterrent to toilet facility use for girls (aIRR = 0.84, 95% CI: 0.71–1.01, *p* = 0.06), but not for boys (aIRR = 1.00, 95% CI: 0.88–1.14, *p* = 0.98). We also found a significant interaction in how facilities with missing shutters were used, based on whether the facility contained urinals or latrines (*p* < 0.01). *Urinal* facilities that didn’t have a shutter had increased use (aIRR = 1.49, 95% CI: 1.13–1.96), whereas *latrine* facilities that didn’t have a shutter had little change in use (aIRR = 0.89, 95% CI: 0.73–1.08), each compared to their counterpart with shutters. Urinal facilities without shutters were designated for primarily for boys (94%). We also observed increased use at newer facilities compared to older ones (aIRR = 1.16, 95% CI: 1.05–1.29). 

**Figure 2:**
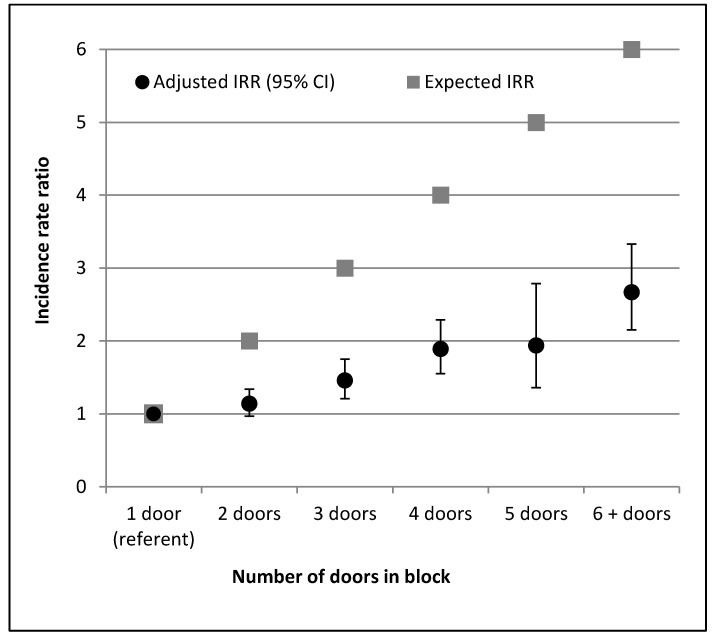
Adjusted IRR and expected IRR comparing the count of pupil uses in a facility with a given number of toilets, to a facility with one toilet, all other variables held constant.

The toilet type and structure also played an important role in facility use. We observed increased use at urinals without shutters (aIRR = 1.86, 95% CI: 1.50–2.32), and decreased use at prefabricated plastic latrines (aIRR = 0.67, 95% CI: 0.52–0.86), each compared to traditional pit latrines. Increasing number of toilets in a block was associated with increased use at that block, however, use did not increase to the degree expected, given the added capacity of the block (Figure 2). 

**Table 4:** Adjusted incidence rate ratio for facility use for each predictor of interest. *

	aIRR^‡^	95% CI	*p*
Toilet facility conditions^ §^			
‘Very dirty’ ^||^	-		
Dirty facility for girls	0.84	0.71–1.01	0.06
Dirty facility for boys	1.00	0.88–1.14	0.98
‘Many flies inside’	1.03	0.89–1.20	0.69
‘No shutter’ ^||^	-	-	
No shutter for urinals	1.49	1.13–1.96	<0.01
No shutter for all other latrines	0.89	0.73–1.08	0.22
Newer age ^¶^	1.16	1.04–1.29	<0.01
Type of toilet facility			
Traditional pit latrine	referent		<0.01
Ventilated improved pit latrine	1.12	0.94–1.33	
Prefabricated plastic latrine	0.67	0.52–0.86	
Uncertain/other **	1.04	0.87–1.23	
Urinal ^||^	-		
Urinals without shutters	1.86	1.50–2.32	<0.01
Urinals with shutters	1.11	0.78–1.60	0.56
Number of toilets per block			
1 toilet	referent		<0.01
2 toilets	1.14	0.97–1.34	
3 toilets	1.46	1.21–1.75	
4 toilets	1.89	1.55–2.29	
5 toilets	1.94	1.36–2.79	
6 or more toilets	2.67	2.15–3.33	

* aIRR compares the count of pupil uses during the 30-minute break between a block with the risk category and a block with the referent category, all other variables held constant. ‡ Model controlled for each of the variables shown in this table, and also the sex designation of the block, the pupil to toilet ratio at the school, latrine coverage in the surrounding community, school enrollment quartiles, wealth index score, geographic district, and study round. The model also accounts for correlation between repeated measures and clustering within schools. § Each of these is a binary variable, where the inverse serves as the referent. || Significant interactions were detected so subgroup specific aIRRs are reported. ¶ Whether the toilet facility was from SWASH+ served as a proxy for newer toilet age. ** The uncertain/other category primarily consists of VIP latrines that were not easily categorized (e.g., missing a fly screen, or a broken pipe).

## 4. Discussion

The purpose of this study was to characterize how a school’s pupil to toilet ratio, and a toilet facility’s characteristics are each associated with toilet use patterns. This is the first study to rigorously characterize many of these relationships, and as such, provides important insights into how to improve pupils’ toilet use and resource allocation for school sanitation.

Our data support the importance of lower pupil to toilet ratios, and quantify the benefits of following guidelines such as those set by the World Health Organization (25:1 for girls, and 50:1 + one urinal for boys) [33] and the Kenyan government (25:1 for girls, and 30:1 for boys) [34]. We also observed increased use of urinals, compared to traditional pits, which is further support for the current WHO guidelines, of including a urinal for boys. The greatest increases in toilet use was seen among schools that easily superseded these guidelines (e.g., <15:1). However, we show that schools with worst ratios, are most likely to benefit, in terms of increased toilet use, from the addition of even a small number of toilets.

In our fully adjusted model, we found some evidence suggesting facility dirtiness may deter girls from toilet use, but not boys. The finding that many pupils are not discriminating which facilities they used based on toilet cleanliness is an important one, as facility cleanliness may be equally, or even more important for pupils’ health and attendance than the pupil to toilet ratio [17]. This finding is also different from previous studies, which detected both a meaningful and statistically significant associations between toilet cleanliness and toilet use for both boys and girls [25,35]. However, our study offers the methodological improvement of control for a number of potential confounders. To replicate these previous studies we performed an unadjusted sub-analysis (data not shown) and were also able to find a statistically significant association, however as we added necessary confounders into the model, this association dissipated. Another important difference between our study and previous studies is that we were able to use observed measures of facility characteristics rather than pupil-reported measures. It is possible that our *observed* measure of cleanliness were different from how pupils actually *perceive* and self-report toilet cleanliness. For example, our enumerators were trained to denote the toilet as dirty based on the presence of dirt, trash, feces, or urine on the floor or walls of the toilet, whereas it is possible that pupils judge toilet cleanliness based on these, but also other factors such as the toilet’s age, structure, or type, which we captured using other variables. 

The number of toilets in the block was an important factor for use. However, we found that increasing the number of toilets in a block does not increase the use proportional to its increased capacity (e.g., doubling the number of toilets does not double pupil use at that block; Figure 2). There are a number of possibilities as to why children may avoid using blocks with more toilets. In other studies, children have reported a number of deterrents to toilet use, including privacy concerns (e.g., insecurity of being heard) [25,26,28], teasing and bullying [25,26,27], and smell [25,26,28], each of which is probably exacerbated by concentrating toilets into larger blocks. 

The amount of privacy required for urination may be different than the amount of privacy required for defecation. This is reflected in our observation that *urinal* facilities that didn’t have a shutter had increased use, whereas *latrine* facilities that didn’t have a shutter had a point estimate reflecting decreased use, although the 95% CI included the null. However, our finding of increased use in urinals without doors is implicitly sex-specific, as 94% of these urinals were actually designated for boys. 

Our study has several limitations. Our study is observational, and therefore has the potential for unmeasured confounding. However, we were able to control for many conceivable confounders. Furthermore our results are biologically plausible and confirm many long-standing, yet untested beliefs. It would also be difficult, and possibly unethical, to randomize some of our exposures of interest, and so observational studies may be the design of necessity. A second limitation is that we were not able to obtain toilet-level pupil use, or the exact reasons for a pupil using a block (e.g., urination, defecation, menstrual hygiene management,* etc.*). We were, however, able to develop valid models that predict block level visits, controlling for the number of toilets within a block. Aggregating latrine-level data, allowed us to observe the relationship between our latrine-level predictors and facility-level use, however, these relationships should be interpreted with care, because when facilities contain several toilets, latrine-level decisions may not always be reflected in what is observed at the block-level (*i.e.*, ecological correlations do not necessarily represent individual correlations). Finally, our results should be generalized carefully. Our study coincided with a ‘light-touch intervention’ trial that provided intervention schools cleaning supplies, and it is not clear what we would have observed had those intervention schools not received any cleaning supplies (e.g., toilets being even dirtier). Our results are most generalizable to similar schools; for example rural, low-resource schools in sub-Saharan African countries.

## 5. Conclusions 

There are a number of factors that play important roles in pupils’ use of school toilets, including pupil to toilet ratio, toilet type, toilet age, and number of toilets in the toilet block, and possibly cleanliness. Cleanliness is of particular interest, as it is likely related to pupils’ exposure to human excrement. This study provides important insights into how to more effectively improve pupil toilet use in schools in developing countries, potentially leading to a better allocation of resources for school sanitation, and to improved health and educational outcomes for children. 

## Appendix

**Table A1:** Inter-rater reliability for the subjective toilet measures.

Variables	Kappa *
‘No shutter’ ^§^	0.88
‘Feces very visible’ ^§,‡^	0.79
‘Most visible urine’ ^§,‡^	0.63
‘Very dirty’ ^§,‡^	0.70
‘Many flies inside’ ^§^	0.63
‘Strong smell inside and outside’ ^§^	0.57

***** We only used variables in our analysis if there was substantial agreement, defined by Landis and Koch to mean a Cohen’s kappa statistic of over 0.6 [30]. ^‡ ^The feces and urine variables were not used in our primary analyses; we chose to use the cleanliness variable instead, which also captures many aspects of a pupils’ exposure to human excrement. ^§ ^A binary variable, where the inverse serves as the referent.
